# Different Means to the Same End: Long-Distance Migrant Seabirds from Two Colonies Differ in Behaviour, Despite Common Wintering Grounds

**DOI:** 10.1371/journal.pone.0026079

**Published:** 2011-10-11

**Authors:** Paulo Catry, Maria P. Dias, Richard A. Phillips, José P. Granadeiro

**Affiliations:** 1 Eco-Ethology Research Unit, ISPA, Lisboa, Portugal; 2 Museu Nacional de História Natural, Lisboa, Portugal; 3 British Antarctic Survey, Natural Environment Research Council, Cambridge, United Kingdom; 4 CESAM, Museu Nacional de História Natural, Universidade de Lisboa, Lisboa, Portugal; University of Regina, Canada

## Abstract

Although seabirds that are trans-equatorial migrants show apparently broad overlap among populations in the non-breeding season, such large-scale pattern may conceal subtle but nevertheless key differences in migratory behaviour. These specializations could reflect adaptation to different environments during the breeding season, carry-over effects from the breeding to the nonbreeding period, or asymmetries in competitive ability of birds of different origin. We compared the migratory and wintering behaviour of Cory's shearwaters *Calonectris diomedea* nesting in Berlengas and in the Selvagens, two colonies in contrasting oceanographic environments, separated by ca. 1200 km. Although no differences were found in winter distribution, there was a marked divergence in timing, route and the use of staging areas during the postbreeding (autumn) migration. Birds from Berlengas typically travelled to oceanic waters in the North Atlantic for an extended stopover, whereas those from Selvagens rarely did so. In the South Atlantic, birds from Selvagens spent more time in flight, perhaps because they had higher energy and nutrient requirements for feather replacement compared to birds from Berlengas, which moult more flight feathers during breeding. Stable isotope analyses of feathers suggested that this variation in activity patterns was unrelated to trophic ecology. Differences in migration routes and stopovers may expose populations to distinct threats, and should be taken into consideration when defining units for conservation purposes and developing appropriate management strategies.

## Introduction

Trans-equatorial migrant seabirds travel huge distances across entire ocean basins, with apparent broad overlap between populations [1]–[3]. The large-scale overlap in nonbreeding distributions amongst populations, may, however, conceal important fine-scale differences in migratory behaviour. These aspects have been little investigated, given the paucity of ringing recoveries from pelagic seabirds and of tracking studies involving sufficient numbers of individual birds of the same species from different colonies.

Birds from colonies close to each other (relative to distant wintering grounds) may display differences in migratory behaviour and wintering ecology due to (1) population specializations arising from local adaptation to breeding environments [4], [5], (2) carry-over effects of conditions experienced during breeding [6], (3) asymmetries in competitive ability of birds of different origins, perhaps caused by 1 and 2, or by other factors, such as small differences in distance to stopover sites and wintering grounds.

Recent developments in technology allow the year-round tracking of pelagic migrants, providing not only geographic position, but also information on activity patterns derived from immersion sensors [7]. Furthermore, studies using stable isotopes in feathers provide insights into their trophic ecology [8]. These methodological advances have not, however, yet been combined with the purpose of comparing the migratory and wintering behaviour of long-distance migrants from different colonies.

Differences between populations improve our understanding not only of migration strategies, but also of demographic changes, as birds may be subjected to distinct threats in different areas, with implications for conservation [9], [10], [11].

The Cory's shearwater *Calonectris diomedea* is a pelagic seabird that nests in the NE Atlantic and Mediterranean and winters in many areas of the Atlantic, mostly in the south [2], [12]. The populations from the Mediterranean *C.* (*d.*) *diomedea* are well differentiated morphologically and genetically from those in the Atlantic *C.*(*d.*) *borealis* [13]. The two (sub) species have also been shown to display considerable differences in migratory behaviour, in terms of their preferred wintering areas [2]. González-Solís et al. [2] also compared the migration of *C.*(*d.*) *borealis* from the Azores and the Canaries, but sample sizes were too small to allow the detection of significant differences in the choice of migration routes or wintering areas.

Here we present tracking data from migrant Cory's shearwater *C.*(*d.*) *borealis* from two colonies – Berlengas, on the Portuguese continental shelf, and the Selvagens, a large offshore colony located north of the Canary islands, where a large-scale study on migration and at-sea behaviour is currently underway [12], [14]. Berlengas represents the main nesting area for *borealis* off continental Europe, which, given major changes in climate and marine systems, may come to represent a significant conservation unit for the taxon. Unlike several others, this population has been increasing in recent decades, was estimated at 850 pairs in 2005 [15] and is now thought to exceed 1000 pairs (M. Lecoq, pers.com.). The Selvagens population (ca. 30,000 pairs) has also been recovering in recent decades from past human depredations [16].

Our main objectives were to: (1) provide the first tracking data on the migration of *C.*(*d.*) *borealis* from continental Europe; (2) compare the wintering areas of Berlengas and Selvagens birds; (3) assess if timing of migration and routes differ between colonies, (4) investigate if birds from the two colonies differ in their behaviour and trophic position in wintering grounds. Knowing that Cory's shearwaters from Berlenga are intermediate in body size and in geographic position of the colony between birds from the Mediterranean and from other Atlantic colonies [13], we expected that Berlenga individuals would perhaps follow an intermediate migratory strategy, choosing wintering areas used by both of those two groups. Furthermore, knowing that birds from Berlenga show greater moult advancement before migration [17], we would expect them to have more spare time, allowing them to adopt different schedules and migratory routes, or a different behaviour in the winter quarters.

## Methods

### Ethics Statement

The deployment of MK7 loggers (see details below) did not take more than 10 minutes and on no occasion did it interfere with reproduction or have visible deleterious effects on study animals. All work was approved by the relevant authorities (Instituto da Conservação da Natureza e da Biodiversidade, for Berlengas, and Serviço do Parque Natural da Madeira, for Selvagens; research permits 107/2006, 116/2007, 333/2007/CAPT).

### Fieldwork and tracking

In 2006/07, we tracked the migratory routes and wintering areas of 11 Cory's shearwaters from Berlengas (39°24′N, 9°30′W) and 25 from Selvagens (30°09′N, 15°52′W) using leg-mounted geolocators (e.g. [17]). Loggers were deployed at the end of the breeding season of 2006 and recovered in the beginning of the next breeding season. Only successful breeders were tracked. Further tracking took place from Selvagens in the subsequent 3 years (2007/08, 2008/09 and 2009/10). The 2006/07 data are used for most comparisons to exclude possible year effects on behaviour, complemented with data from subsequent seasons to better assess the general preference for wintering regions of Cory's shearwaters from Selvagens.

Geolocators (mk 7 model, mass approximately 3.6 g, developed by British Antarctic Survey, Cambridge) provide two positions per day based on light levels (one at local midday and other at local midnight), with an accuracy of approximately 186±114 km [18]. Following download, light data were analysed using *TransEdit* software (see [12] for more details). The final part of the return migration of many Cory's shearwaters coincides with spring equinox when latitude estimation is unreliable. Arrival dates could nevertheless be determined based on longitudes, given the obvious easterly movement of birds as they approach the colony [12]. The onset of the postbreeding migration (i.e., the departure date from colony) was difficult to determine in a few cases for birds from Selvagens, given the low accuracy of the geolocation method and because some birds remain in the Canary current for a period before migration. Therefore, we considered that birds had left the colony when all subsequent positions were outside a 200 km radius from Selvagens. We also calculated an additional and easily determined measure of the beginning of the southward journey for comparing between colonies – the date at which birds crossed the 10^th^ north parallel. The date of departure from the wintering areas was defined as the onset of a clear northward directional movement.

### Stable isotope and statistical analyses

Upon recapture of the study birds for logger recovery, we cut a small section of primary 8, which is known to be grown in the wintering grounds [17], [19] for stable carbon (δ^13^C) and nitrogen (δ^15^N) isotope analyses. Sub-samples of 1.0±0.1 mg of finely cut pieces of feather were weighed into 8×5 mm tin capsules and combusted at 1000°C in a Euro EA Elemental Analyser. Resultant CO_2_ and N_2_ gases were analysed using a continuous-flow isotope ratio mass spectrometer IsoPrime (MicroMass). Replicate measurements of laboratory standards showed measurement errors of ±0.1‰ and ±0.3‰ for stable carbon and nitrogen isotope measurements, respectively. Laboratory standards were “Methionine OAS B2045” and “L-Cystine OAS B2035”, calibrated and verified every six months with the standards IAEA-CH6, IAEA-CH7, IAEA-600 e IAEA-N1 from the International Atomic Energy Agency, Vienna. Quality control samples (one of our randomly chosen samples) were run in triplicate before and after each sequence, and feather samples were run interspersed with laboratory standards (3 standards every 30 unknowns).

One of the loggers recovered from a bird at Berlengas ceased functioning before arrival at the wintering grounds, and hence only provided information on the postbreeding migration. Comparisons of timing and routes taken during the postbreeding migration only included individuals that wintered in the South Atlantic.

Activity patterns during the winter period (namely the percentage of time spent on sea surface and the number of landings per hour) were derived from saltwater immersion data (wet/dry), registered by the geolocators with a 3 s precision. In addition, a night flight index was calculated as the percent of total time spent in flight that occurred in darkness, divided by the relative duration of the dark period in each day. This is a measure of the amount of flight undertaken during darkness rather than daylight, corrected for the overall duration darkness relative to daylight, i.e., a value of 1 corresponds to an allocation of the flight time during daylight and darkness proportional to the duration of each phase.

Comparisons of the date of departure, the date crossing the 10^th^ North parallel and the date of arrival at the colony were made using Mann-Whitney tests. Comparisons of the timings of other events, of mean activity indices per individual, and of isotope values were made using two-way ANOVAs, with wintering area (Benguela *vs.* central South Atlantic) and nesting colony (Berlengas *vs.* Selvagens) as fixed factors.

## Results

**Figure 1:**
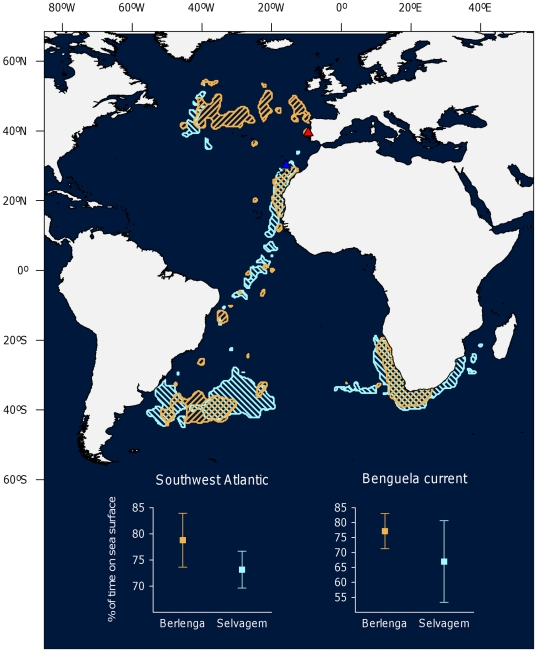
Non-breeding distribution of Cory's shearwaters from Berlengas (n = 10, red shading) and from Selvagens (n = 25, blue shading) and activity levels on two main wintering grounds. Shadings represent the 95% kernel density contours for each group of study birds tracked with geolocators in 2006/07. Triangles indicate colony locations. Note that birds from Berlengas spent more time resting on the sea surface in each of the two main wintering areas.

The non-breeding distributions of Cory's shearwaters from Berlengas and Selvagens are broadly similar between them (Fig. 1). Sixty percent (n = 10) of the birds from Berlengas wintered in the Benguela-Agulhas region in 2006/07, which compares with 52% (n = 25) of birds from Selvagens in the same year, and 64% (n = 67 individuals) in 2006/07 to 2010/11. Other areas used by individuals from Berlengas were also frequented by birds from Selvagens: the central South Atlantic (30% of the birds from Berlengas and 32% of the birds from Selvagens) and Canary current (Berlenga: 10%; Selvagens: 4%).

**Figure 2:**
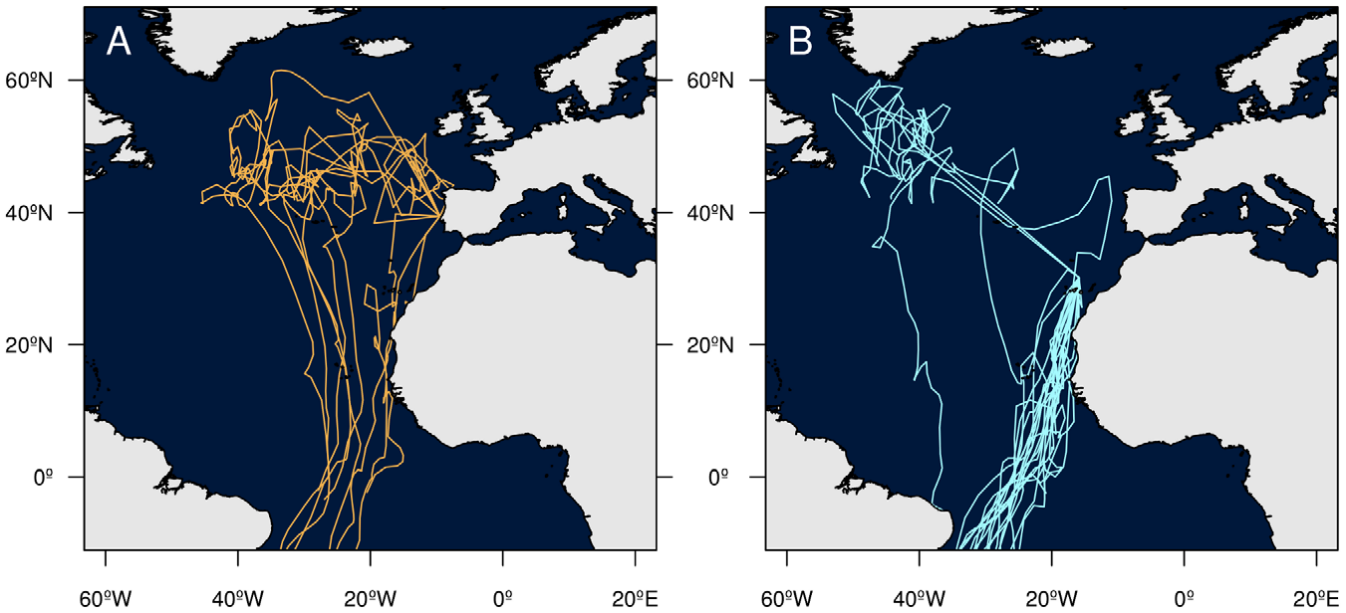
Post-breeding (autumn 2006) migration tracks of Cory's shearwaters. Tracks from (A) Berlengas and from (B) Selvagens that wintered in the South Atlantic. Note that while most individuals from Berlengas made extensive excursions into North Atlantic pelagic waters, those from Selvagens mostly traveled directly from the colony area to the South Atlantic.

In 2006, departure dates for the postbreeding migration were earlier at Berlengas than at Selvagens (Table 1). At least 7 out of 10 birds from Berlengas made a large detour during the southerly migration, to spend on average 26±10 days in offshore North Atlantic waters, whereas 20 out of 23 of birds from Selvagens took a more direct route to the South Atlantic (Fig. 2), a difference that was statistically significant (Fisher's Exact Test P = 0.002). Indeed, the difference may be larger than suggested by these figures, because the remaining three birds from Berlengas departed close to the equinox, and although their trajectories could not be mapped with certainty, all nevertheless appeared to make long detours in the North Atlantic. Other migration routes and corridors were generally similar between the two colonies (not shown). In subsequent years, the Selvagens birds made little use of the North Atlantic waters, with 14 out of 15 individuals going directly to the south Atlantic in 2007/08, 5 out of 8 in 2008/09 and 15 out of 15 in 2009/10 (these figures do not include repeat migrations by the same individuals).

**Table 1:** Timing of migration of birds from Berlengas and Selvagens in winter 2006/07.

	Berlengas	Selvagens	Statistical comparison
Departure date from colony	22 Oct±11 d	7 Nov±12 d	Mann-Whitney Test, U = 38.5; P = 0.003
	(n = 10)	(n = 23)	
Date of transit south across 10° N	19 Nov±7 d	18 Nov±13 d	Mann-Whitney Test, U = 80.0; P = 0.335
	(n = 9)	(n = 23)	
Arrival at the wintering area:			Col.: F = 0.03; p = 0.871, Wint. area: F = 0.277; p = 0.604
Benguela	6 Dec±11 d	5 Dec±11 d	
	(n = 6)	(n = 10)	
South Atlantic	10 Dec±9 d	7 Dec±9 d	
	(n = 3)	(n = 8)	
Departure from wintering area:			Col: F = 3.52; p = 0.072, Wint area: F = 0.08; p = 0.780
Benguela	20 Feb±5 d	19 Feb±9 d	
	(n = 6)	(n = 10)	
South Atlantic	27 Feb±8 d	15 Feb±7 d	
	(n = 3)	(n = 8)	
Return to the colony:	19 Mar±10 d	12 Mar±10 d	Col: F = 3.09; p = 0.091, Wint area: F = 0.028; p = 0.888
	(n = 9)	(n = 18)	
Benguela	16 Mar±10 d	15 Mar±10 d	
	(n = 6)	(n = 10)	
South Atlantic	27 Mar±8 d	09 Mar±9 d	
	(n = 3)	(n = 8)	

Activity patterns in the wintering grounds in the South Atlantic differed between birds from different colonies (% time in the water), and between wintering areas (number of landings per hour; night versus daylight activity) (Fig. 1, Table 2). δ^13^C values differed between wintering areas, but not between birds of different populations, whereas δ^15^N showed no significant variation according to origin or wintering area (Table 2).

**Table 2:** Activity patterns and stable isotope levels in primary 8 of Cory's shearwaters from two colonies wintering either in Benguelas or in the central South Atlantic.

	Berlengas		Selvagens		Comparison
	Benguela (n = 6)	Cental South Atlantic (n = 3)	Benguela (n = 10)	Central South Atlantic (n = 8)	
% of time on sea surface	77.1±5.9	78.8±5.2	66.9±13.7	73.1±3.5	Col: F = 4.56; p = 0.04, Wint area: F = 1.75; p = 0.20
Landing rate (landings per hour)	4.5±1.0	5.8±1.0	4.3±0.8	7.1±2.1	Col: F = 1.19; p = 0.29, Wint area: F = 18.76; p = 0.0002
Night flight index	0.4±0.1	0.9±0.1	0.5±0.2	0.8±0.2	Col: F = 0.66; p = 0.42 Wint area: F = 24.16; p<0.0001
δ^13^N in P8	15.09±0.92	15.28±0.95	15.45±0.36	14.56±1.36	Col: F = 0.09; p = 0.76, Wint area: F = 1.97; p = 0.17
δ^13^Cin P8	−15.22±1.21	−16.16±0.32	−14.96±0.59	−16.12±0.31	Col: F = 0.07; p = 0.93, Wint area: F = 15.49; p = 0.0007

## Discussion

This study presents the first tracking data for *borealis* Cory's shearwaters nesting near continental Europe, and is the first published comparison of wintering site selection, timing of migration and choice of route, activity patterns and feeding ecology (based on stable isotope analysis) of birds from different breeding populations in a long-distance migrant seabird.

Both morphologically (in body size) and geographically, Cory's shearwaters from Berlengas are intermediate between other *borealis* populations and the nominate birds in the western Mediterranean (those tracked by González-Solís et al. [2]). In evolutionary terms, however, birds from Berlengas are tightly clustered with other *borealis* and well separated from the *diomedea* subspecies [13], [20]. It has been suggested that body size (through its influence on wing-loading) is a key determinant of habitat selection by dynamic-soaring, pelagic seabirds such as Cory's shearwaters [21], [22]. In this context, it is noteworthy that the choice of wintering areas of birds from Berlengas resembles much more closely that of populations from the Atlantic, and clearly diverges from birds from Mediterranean colonies [2], [23]. This suggests that taxonomic influences, rather than geographical location or differences in body size *per se*, best account for the diversity of migratory behaviour previously reported for Cory's shearwaters [2]. Recent research suggests that the Atlantic and the Mediterranean forms of Cory's shearwater are probably best regarded as separate species, with an estimated divergence time of 1 million years, despite subsequent limited gene flow [13], [20]. Hence, differences in wintering ranges are not unexpected. Indeed, González-Solís et al. [2] and Ristow et al. [23] present results indicating that it is unusual for Cory's Shearwaters from Mediterranean colonies to migrate as far as the South Atlantic, which is in clear contrast to birds from Macaronesia and Berlengas ([12], this study).

Our study is novel in comparing two populations, using adequate sample sizes, to reveal subtle, but nevertheless striking differences in migratory behaviour of a pelagic long-distance migrant. Berlengas and Selvagens are separated by <1200 km, which is a relatively short distance when considering that many Cory's shearwaters migrate >30,000 km between nesting seasons ([2], own unpubl. data). Despite this, there is a marked divergence in strategies adopted during the postbreeding (autumn) migration between these two populations, even though there is no difference in the reproductive schedule [24]. Birds from Berlengas departed from the waters around the colony almost two weeks earlier and, in the great majority of cases, took a large detour to previously unknown staging areas located in offshore North Atlantic waters, particularly northwest and north of the Azores. Far fewer birds from Selvagens took this route, instead travelling directly to the South Atlantic or, in a few cases, spending an initial period after their last colony visit in the Canary upwelling region. It should be noted that the staging area in the northwest Atlantic is no closer to Berlengas than to Selvagens (approximately 2,300 km from both colonies), and so relative proximity cannot be a suitable explanation for the difference in strategies. Another possibility would be if prey availability were lower in the waters around Berlengas, that birds from there had a greater need to gain body condition in a suitable staging area in preparation for the long crossing of the unproductive tropical Atlantic. However, the area around Berlengas, on the Portuguese shelf, is no less productive than the deep waters surrounding the Selvagens, and indeed, Cory's shearwaters seem to have greater difficulty in finding food at the latter site [24]–[26]. How conditions in the Canary current compare with those on the Portuguese shelf and in the Atlantic staging area in autumn is uncertain. Hence, the reason why more shearwaters from Selvagens and Berlengas, once freed from reproduction, do not move south at least initially to shelf and shelf break waters of the nearby Canary upwelling is unknown, but presumably relates to relative resource availability and levels of intra- and inter-specific competition. It is interesting to note that Cory's shearwaters from these two sites adopt different strategies in flight feather moult, with many more birds replacing feathers at the end of the nesting season (September–October) in Berlengas than in Selvagens, presumably because better foraging conditions at the former site allow a greater moult-breeding overlap [17].

Recently, much interest has arisen from the demonstration that events during the non-breeding period may affect condition and performance during breeding, the so called carry-over effects [27]. This is a difficult subject to study, and most attention has been devoted to possible effects of wintering habitat on the subsequent breeding season. However, there is no reason why events on the breeding grounds should not similarly affect birds in their wintering areas, perhaps influencing their condition, behaviour and survival [6]. A striking result of our study was that Cory's shearwaters nesting on Berlengas (a site where food availability allows a greater progression of moult before migration [17]) show reduced levels of flight (spending more time resting in the water) and hence presumably lower energy expenditure than birds from Selvagens in shared winter quarters. Moult is a demanding process [28], and it is plausible that individuals that have more feathers left to moult in the winter quarters (those from Selvagens) need to be more active, to gather the necessary energy and nutrients needed for feather growth.

Isotope composition of feathers moulted in winter quarters were similar for birds of the two colonies, and hence the reported difference in activity levels is probably not accounted for birds from different colonies (and body sizes) targeting different types of prey. We note, however, that similar isotope values do not necessarily imply a similar diet, although that is the most likely scenario. An experimental approach would be recommended, to further assess this new hypothesis that the degree of moult progression in the breeding grounds affects bird behaviour in distant wintering areas.

It is also interesting to note that, irrespective of the colony of origin, individuals adjusted their activity patterns to the areas in which they wintered, presumably as a response to local resource availability patterns, which confirms that Cory's shearwaters are flexible in several dimensions of their migratory behaviour, as reported here and elsewhere [12]. Despite variation in moult strategies, postbreeding migration routes and activity levels during winter, there was no significant difference in the mean dates that Cory's shearwaters from Berlengas and Selvagens arrived back at their nesting colonies, hence reaching the same end by different means.

The difference in migration strategies between birds from different populations should also encourage their conservation as separate management units, as they may not be equally resilient to predicted changes in future climate and marine ecosystems.
